# Immune-related gene-based model predicts the survival of colorectal carcinoma and reflected various biological statuses

**DOI:** 10.3389/fmolb.2023.1277933

**Published:** 2023-10-18

**Authors:** Zhengchun Kang, Bingchen Chen, Xiuzhu Ma, Feihu Yan, Zhen Wang

**Affiliations:** Department of Colorectal Surgery, Changhai Hospital, Naval Medical University, Shanghai, China

**Keywords:** colorectal cancer, immune, prognosis, model development, gene expression

## Abstract

**Bakcground:** Prognosis of colorectal cancer (CRC) varies due to complex genetic–microenviromental interactions, and multiple gene-based prognostic models have been highlighted.

**Material and Method:** In this work, the immune-related genes’ expression-based model was developed and the scores of each sample were calculated. The correlation between the model and clinical information, immune infiltration, drug response and biological pathways were analyzed.

**Results:** The high-score samples have a significantly longer survival (overall survival and progression-free survival) period than those with a low score, which was validated across seven datasets containing 1,325 samples (GSE17536 (N = 115), GSE17537 (N = 55), GSE33113 (N = 90), GSE37892 (N = 130), GSE38832 (N = 74), GSE39582 (N = 481), and TCGA (N = 380)). The score is significantly associated with clinical indicators, including age and stage, and further associated with PD-1/PD-L1 gene expression. Furthermore, high-score samples have significantly higher APC and a lower MUC5B mutation rate. The high-score samples show more immune infiltration (including CD4^+^ and CD8^+^ T cells, M1/M2 macrophages, and NK cells). Enriched pathway analyses showed that cancer-related pathways, including immune-related pathways, were significantly activated in high-score samples and that some drugs have significantly lower IC_50_ values than those with low score.

**Conclusion:** The model developed based on immune-related genes is robust and reflected various statuses of CRC and may be a potential clinical indicator.

## Introduction

As the fifth most common cancer, it is estimated that 376,300 new colorectal cancer cases and 160,600 related deaths occurred in China in 2015 (Chen et al., 2016). Despite the reports regarding the clinical significance of clinical indicators and genomic features, their clinical utilization and performance are still not satisfactory (Sveen et al., 2020; Yamamoto et al., 2021; Martelli et al., 2022). Recently, genome-wide screening-based prognostic models have been emphasized, due to their robustness in reflecting the multiple features of various types of cancers for classification, prognosis, and therapy guidance (Yue et al., 2021; Hou et al., 2022; Zheng et al., 2022), and some models have been recommended under ASCO’s guidance (Andre et al., 2022; Martelli et al., 2022) across various types of cancers. For example, Likun et al. developed a LASSO-based model with hypoxia immune-related lncRNAs and tested its clinical relevance, including immune cell infiltration and drug sensitivity (Luan et al., 2022). Songtao et al. developed a model based on ferroptosis-related genes (FRGs) and validated it with another cohort (Du et al., 2022), and the knocking down of some genes affects the phenotypes of colorectal cancer cell lines. Another report used immune-related genes and developed a model for prognosis (Wang et al., 2020).

Immune escape is one of the hallmarks of cancer, while a lot of genes and cells participate in this process (Liu et al., 2020; Schmitt and Greten, 2021). Even genes that regulate metabolism may participate in this process (Zhang et al., 2021). Their immune status not only influences the outcomes of immune therapy but also those of chemotherapy and targeted therapy (Woolston et al., 2019; Picard et al., 2020; Majidpoor and Mortezaee, 2021). However, these models lack sufficient validation datasets. The robustness of multiple gene-based models is a result of reflecting the various statuses of cancer cells, while a lack of sufficient validation datasets weakens the prognostic value of the model. In addition, the biological aspect that the models reflect is not deeply discussed and the function of the genes in the model was not assayed.

In this vein, an immune gene-based model was developed in TCGA cohort and was validated in six other validation cohorts. In addition, the clinical and genetic (including mutation, CNA, and DNA methylation) associations were analyzed. Enriched Gene Ontology (GO) and KEGG pathways were identified using GO and gene set enrichment analyses (GSEA), respectively. The immune status difference between the subtypes was assayed, and putative drug sensitivity analyses between subtypes were also carried out. The expression levels of the genes in the model were also assayed using qPCR and Western blot, and the functions of the genes used in cell proliferation and migration were verified. As a result, the genes in the model are associated with clone formation, proliferation, migration, and invasion in CRC and the model is shown to reflect multiple statuses of colorectal cancer.

## Results

### Candidate gene identification and model development

The gene expression data and clinical information were retrieved from public databases, including TCGA dataset (N = 380) and GSE39582 (N = 481), and a correlation between gene expression and the clinical outcome (including overall survival and progression-free survival) was evaluated using both Cox univariate regression and group comparison by dividing the samples into high-expression and low-expression samples, according to each gene’s median expression value. The genes significantly associated with both progression-free survival and overall survival were identified as list1. Furthermore, immune-related genes were retrieved from MSigDB as list2. Only genes in both list1 and list2 were identified as candidate genes. Finally, there were 14 genes being identified as candidate genes. To narrow down the panel, information redundancy was removed, and the potential clinical utilization was facilitated; the combination of these 14 genes was enumerated; the performance of each combination was evaluated in both GSE39582 and TCGA datasets, and a combination of 11 genes was selected as the final panel. Afterward, the Cox multivariate regression model was developed using the gene expression value of the overall survival information in TCGA dataset. The scoring model was calculated as follows: Score = (0.19745 × PRG3) + (−0.05706 × PAK1) + (0.15915 × LTB4R) + (−0.30012 × ICOS) + (−1.05201 × SFPQ) + (0.17938 × LEP) + (−0.15633 × VIM) + (0.14018 × SSC5D) + (−0.00910 × CASP6) + (0.10686 × SLC11A1) + (0.13690 × UNG).

**FIGURE 1:**
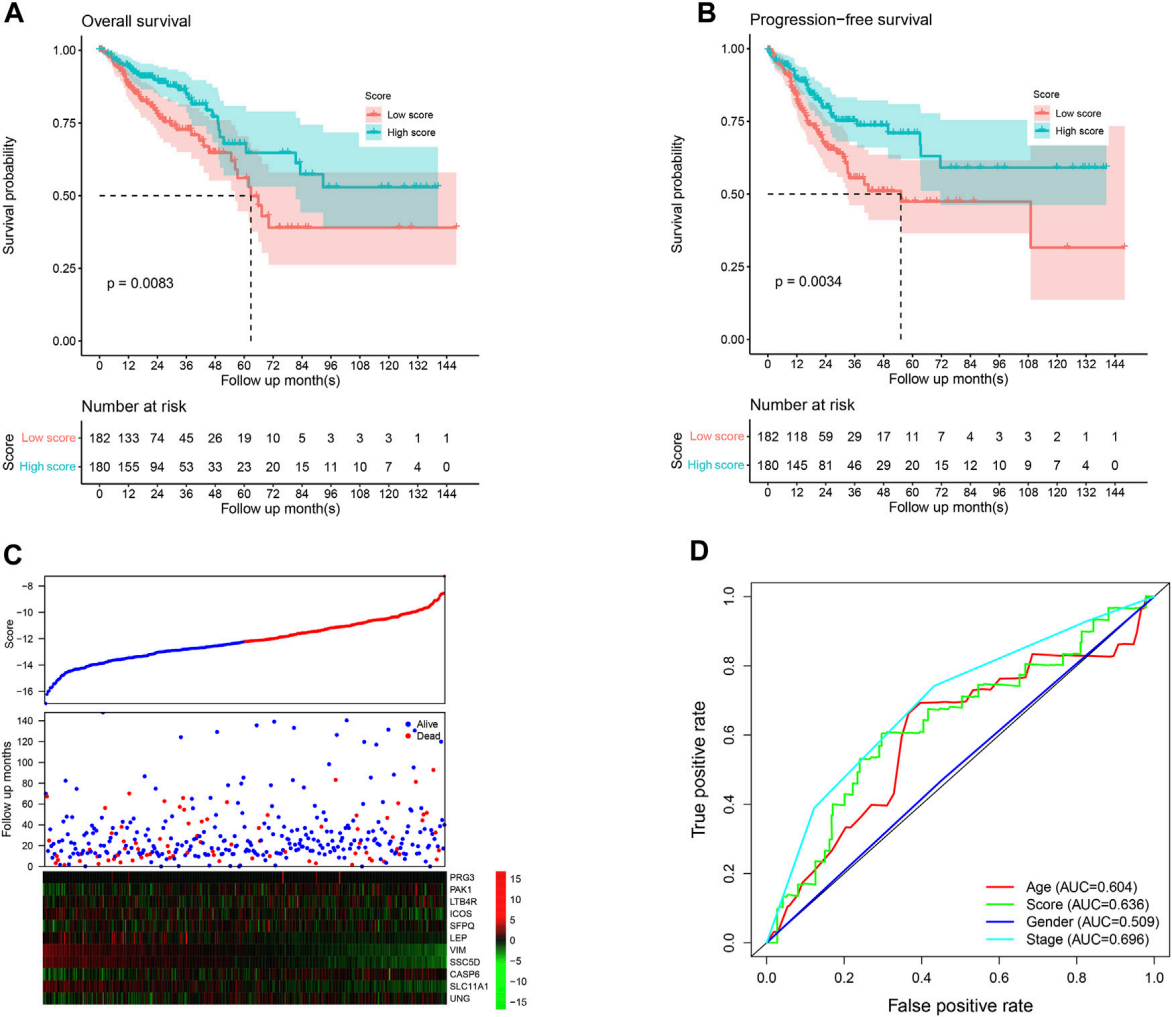
Performance of the model in the training set, TCGA. The overall survival **(A)** and progression-free survival **(B)** in the high-score group are significantly higher than the low-score samples. The high-score samples showed high expression of tumor suppressor genes and low expression of oncogenes **(C)**. The samples were ranked according to the score value (low to high), and survival information and gene expression are also visualized using a heatmap. Survival ROC curve of clinical information and the score is visualized **(D)**.

The score value of each sample was calculated, and patients in TCGA dataset were divided into the high-/low-score group; the overall survival difference between these groups was evaluated. High-score samples have prolonged the survival time compared to low-score samples (Figure 1A; *p* <0.01). Consistent with this, the progression-free survival of high-score samples also has a better survival than those with a low score (Figure 1B; *p* <0.01). The three-year survival receiving operating characteristic (ROC) curve showed that the score shows good performance in predicting the clinical outcome of CRC patients in TCGA dataset (Figure 1D). These genes in the model were used to construct a protein–protein interaction network in the STRING database, but most genes were not connected, which is reasonable since these genes were considered to be complementary for a prognosis (Supplementary Figure S1).

### Model verification

**FIGURE 2:**
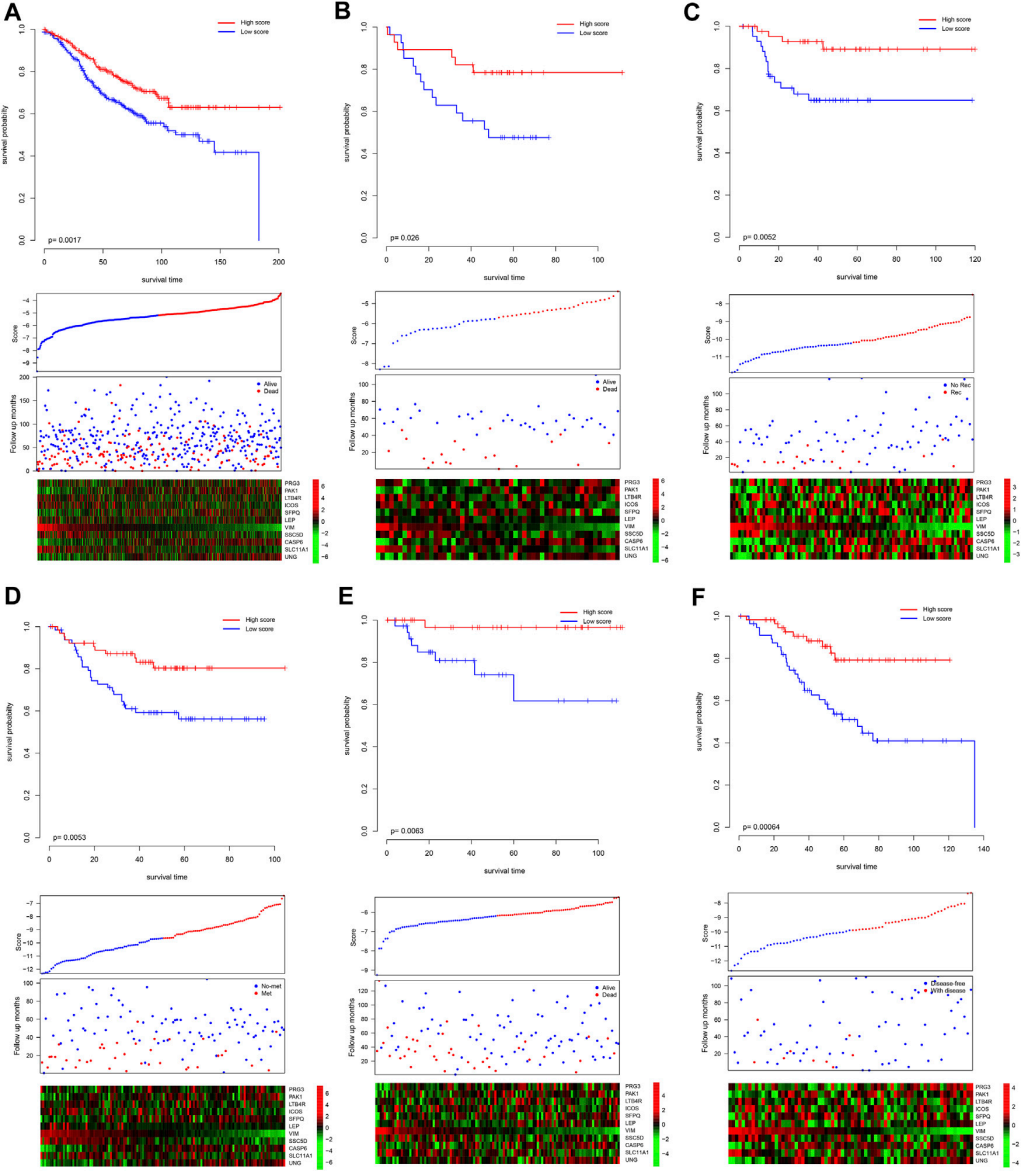
Validation performance of the model. The score of each sample was calculated, and the samples in each dataset were divided as high-/low-score groups, according to the median value of each dataset. The survival difference was analyzed among the groups in GSE39582 **(A)**, GSE17537 **(B)**, GSE33113 **(C)**, GSE37892 **(D)**, GSE38832 **(E)**, and GSE17536 **(F)**. The upper panel showed the survival difference, and the bottom panel showed the detailed score, survival, and gene expression information. Recurrence-free survival, metastasis-free survival, and disease-free survival. Rec and no-rec represent recurrence and no-recurrence, respectively; met and no-met indicate metastasis and no-metastasis, respectively; disease-free and with disease represent the disease-free survival and with the disease, respectively.

Since the model was developed using TCGA dataset, the good performance of the model may result from the overfit. The model was further validated in independent datasets. Scores of each sample were calculated according to the formula listed previously, and the samples in each dataset were divided into high-/low-score groups. As shown in Figure 2A, consistently with TCGA dataset, the high-score sample showed a better survival rate in GSE39582. Notably, despite the fact that the model was not developed in the GSE39582 dataset, the candidate gene selection considered the performance of GSE39582, and five additional independent datasets, namely, GSE17536, GSE17537, GSE33113, GSE38832, and GSE37892, were used for validation. As expected, the survival time of the samples with high scores is significantly longer than those with a low score (Figures 2B–F). Furthermore, high-score samples tend to have a low death rate, high expression of tumor suppressor genes, and low expression of oncogenes, as the training dataset. Collectively, these results indicate that the model is robust across the datasets and platforms that measure gene expression.

### Association between the score and clinical information

**FIGURE 3:**
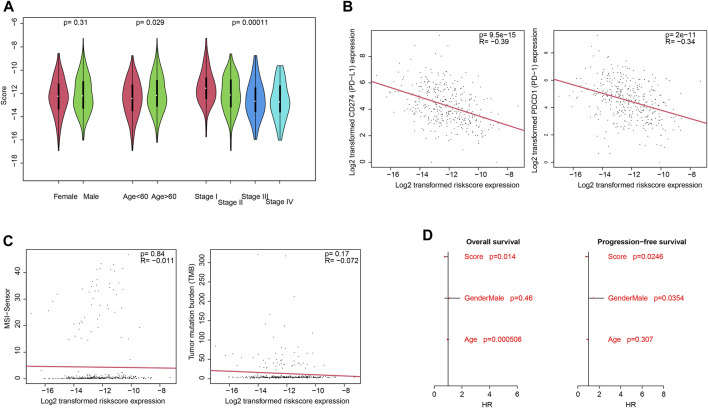
Clinical associations with the score. Association between the score and clinical categories **(A)**, PD-1/PD-L1 gene expression **(B)**, and MSI/TMB **(C)** were analyzed. Cox multivariate regression showed that the score is an independent and significant indicator, for both overall survival and progression-free survival **(D)**.

Clinically, pathological information is widely used for prognosis and therapy decisions. Thus, the correlations between the score and clinical information were evaluated. As shown in Figure 1A, the score value is independent of gender but is significantly associated with the age and pathological stage. For immune infiltration and function, PD-1 and PD-L1 activities are an important indicator, and the correlation between the score and PD-1/PD-L1 was also estimated. As a result, the score value is significantly and negatively associated with PD-1 and PD-L1 mRNA expression (Figure 3B; *p* <0.0001; R = −0.39 and −0.34, respectively), which is consistent with the previous results. However, the score value is not significantly correlated with the tumor mutation burden (TMB) and the MANTIS MSI score (Figure 3C). The Cox multivariate regression showed that the score is significantly and positively associated with a better overall and progression-free survival (Figure 3D; *p* <0.05), suggesting that it is an independent and valuable indicator for survival.

### Genetic signature of high-score samples

**FIGURE 4:**
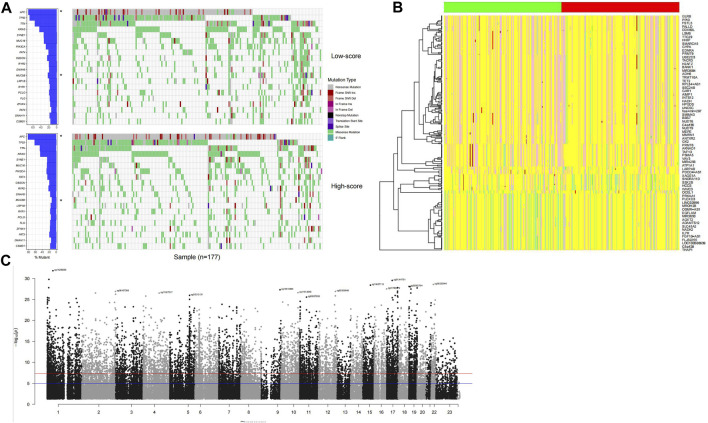
Genomic signature of high-score samples. The high-score samples have a significantly higher APC mutation rate and a lower MUC5B mutation rate **(A)**, compared to the low-score samples. Copy number variation signature was observed **(B)**; the green and red column bars indicate low- and high-score samples, respectively, and specific DNA methylation distributed across the genome **(C)**.

The genetic signatures of the model were analyzed, including mutation, copy number variation (CNV), and DNA methylation. By comparing high-/low-score samples, it is found that the APC gene mutation rate of high-score samples is significantly higher than that of low-score samples, while MUC5B values are lower in high-score samples (*p* <0.05; Figure 4A). A specific signature of copy number variations was also detected in high-/low-score samples (Figure 4B). The DNA methylation status of the distributed high-score samples is dispersed across the genome (Figure 4C), which is consistent with the gene distribution in the model.

### Pathways that the score reflected

**FIGURE 5:**
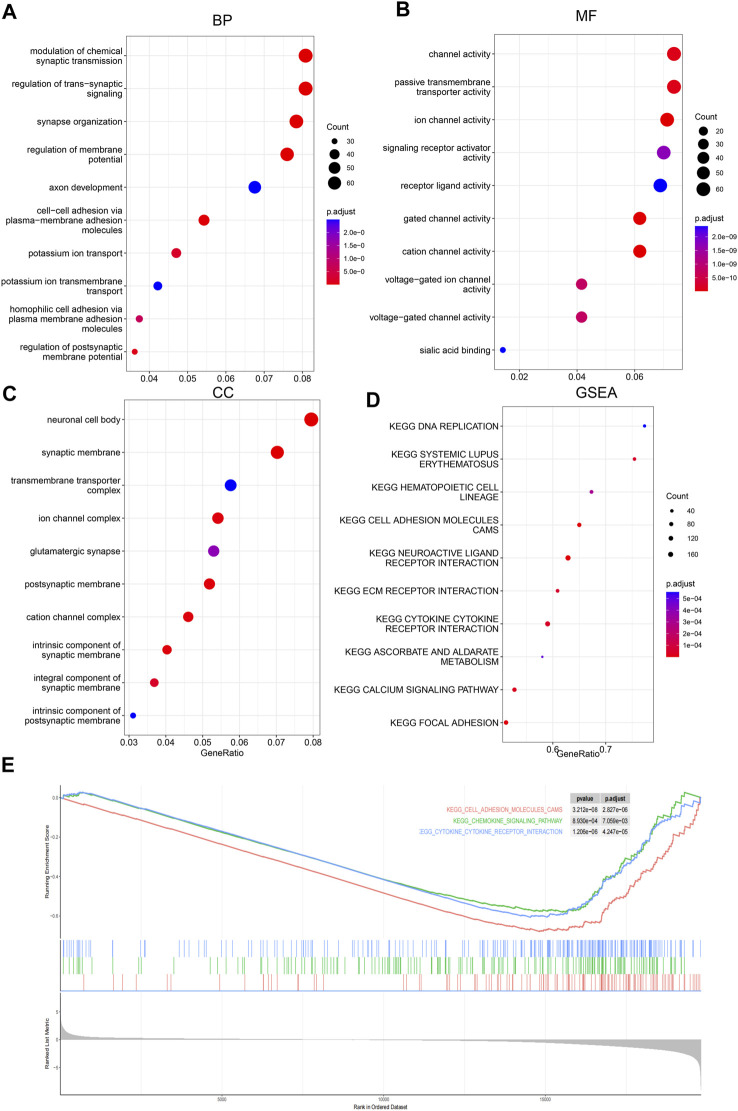
Gene Ontology and GSEA. Differential genes between low-/high-score samples were identified, and Gene Ontology analyses were carried out; cancer-related biological process (BP), **(A)**, molecular function (MF), **(B)** and cellular component (CC), and **(C)** were enriched. Gene set enrichment analyses showed that cancer-related pathways **(D)** were enriched, including immune-related pathways **(E)**.

To investigate the potential pathways and the biological function/process that the score may reflect, Gene Ontology and gene set enrichment analyses were carried out to identify the significantly differentially activated pathways. As shown in Figures 5A–D, the tumor-related process was significantly enriched. Consistently, KEGG pathways, including cell adhesion, the chemokine signaling pathway, and cytokine–cytokine receptor pathways, were significantly enriched in low-score samples (Figure 5E). Collectively, these results suggest that they reflect various statuses of tumor samples, including immune-related pathways.

### Immune infiltration and the score

**FIGURE 6:**
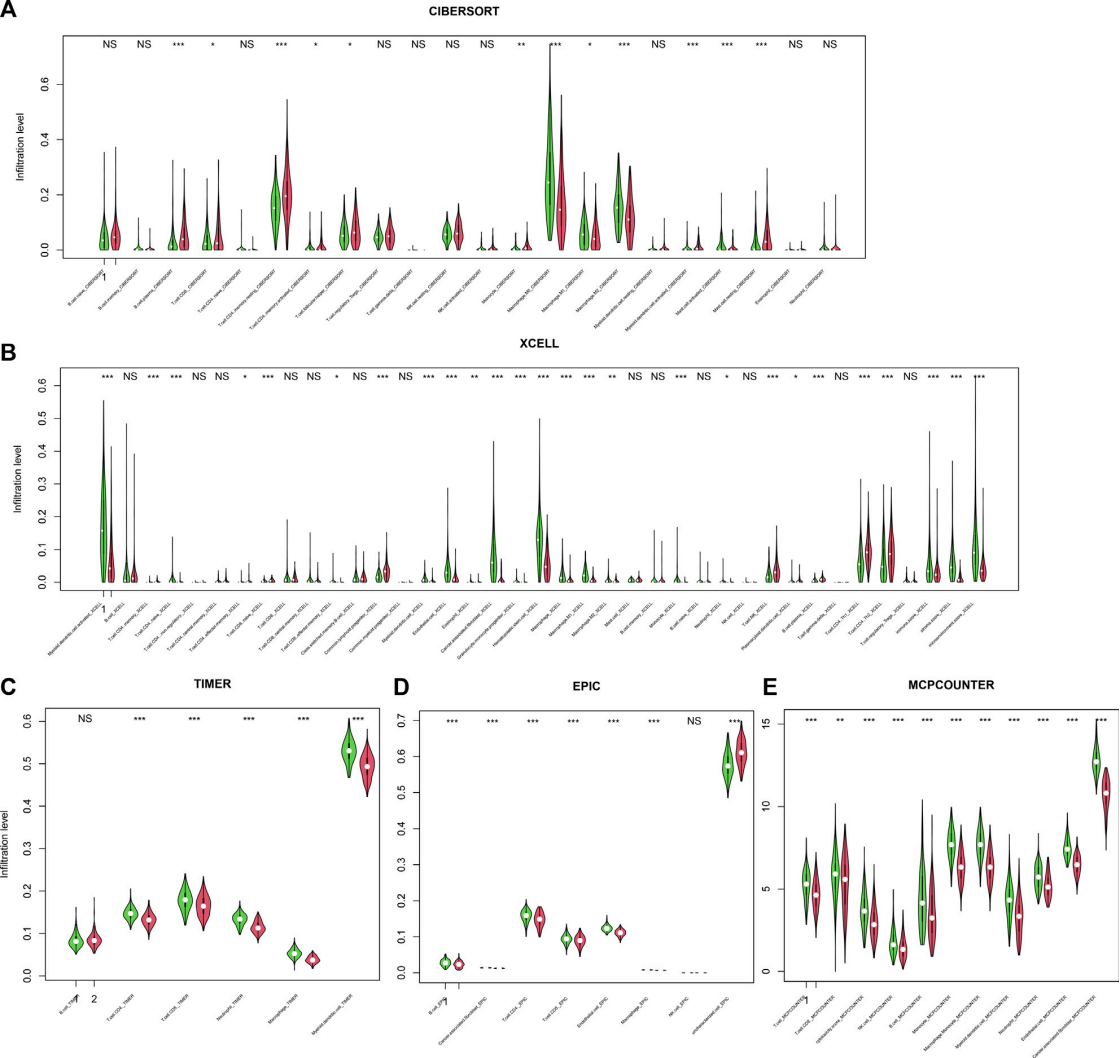
Infiltration and score. The score is significantly associated with immune cell infiltration, regardless of the calculation algorithm, including CIBERSORT **(A)**, xCELL **(B)**, TIMER **(C)**, EPIC **(D)**, and MCP-counter **(E)**.

Immune escape is one of the hallmarks of cancer, and immune infiltration is necessary for an immune response. Thus, the relationship between infiltration and the score was assessed. To comprehensively assess the immune infiltration status, algorithms that calculate infiltration abundance, including CIBERSORT, xCELL, TIMER, EPIC, and MCP-counter, were used for immune infiltration estimation. As a result, important immune-related cells, including CD4^+^, CD8^+^, Th^+^, and CD4^+^ memory cells; M1/M2 macrophages; NK cells; and dendritic cells, were differentially infiltrated between high-/low-score samples (Figures 6A–E), suggesting that the score reflected the immune status of CRC.

### Putative drug sensitivity and the score

**FIGURE 7:**
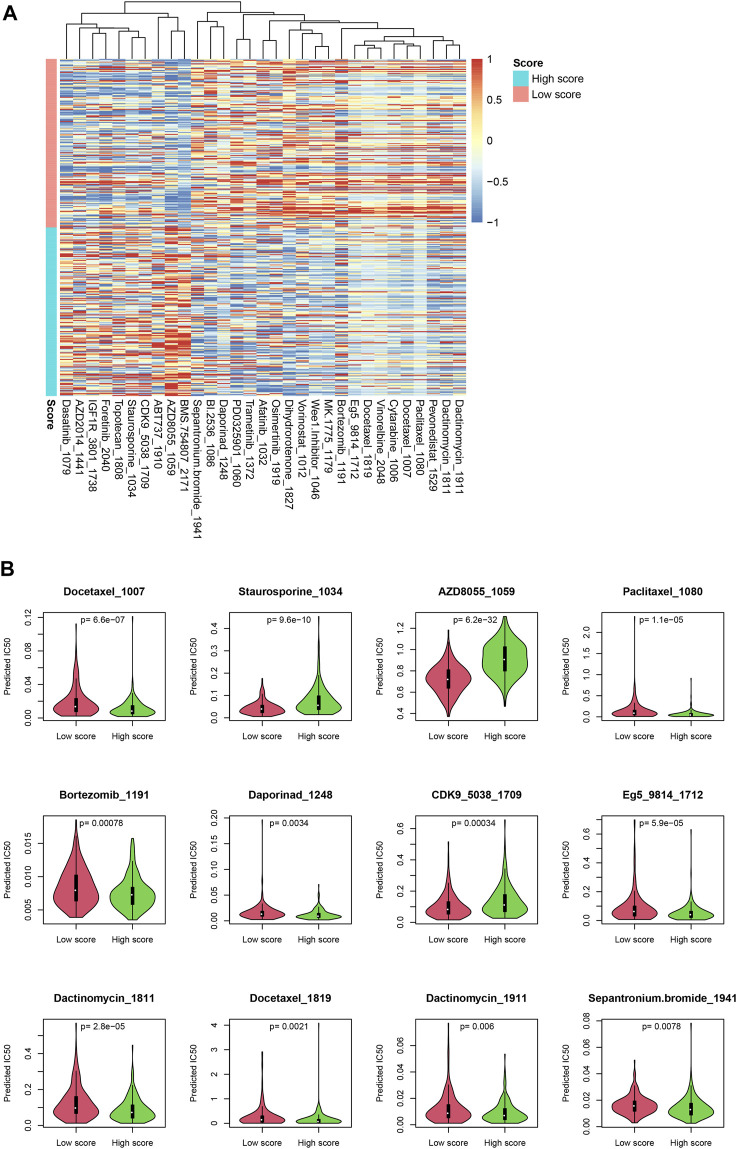
Drug sensitivity difference between low-/high-score samples. The drugs with significantly different IC_50_ values were identified **(A)**. Detailed statistical information on some drugs is also shown **(B)**.

Since the score reflected multiple statuses of CRC, we estimate whether the score could be an indicator for drug resistance/sensitivity. The IC_50_ value of each sample was calculated according to the “oncopredict” algorithm, and the relationship between the score and the putative IC_50_ value was evaluated. As shown in Figure 7A, the predicted IC_50_ values in high-score samples were significantly lower than those in low-score samples, for a lot of drugs, including docetaxel and paclitaxel, while other drug levels were significantly higher in high-score samples, including topotecan and dasatinib (Figure 7B), which may suggest that the score may be used for making a therapy decision, after careful validation.

### Nomogram for the score and clinical information

**FIGURE 8:**
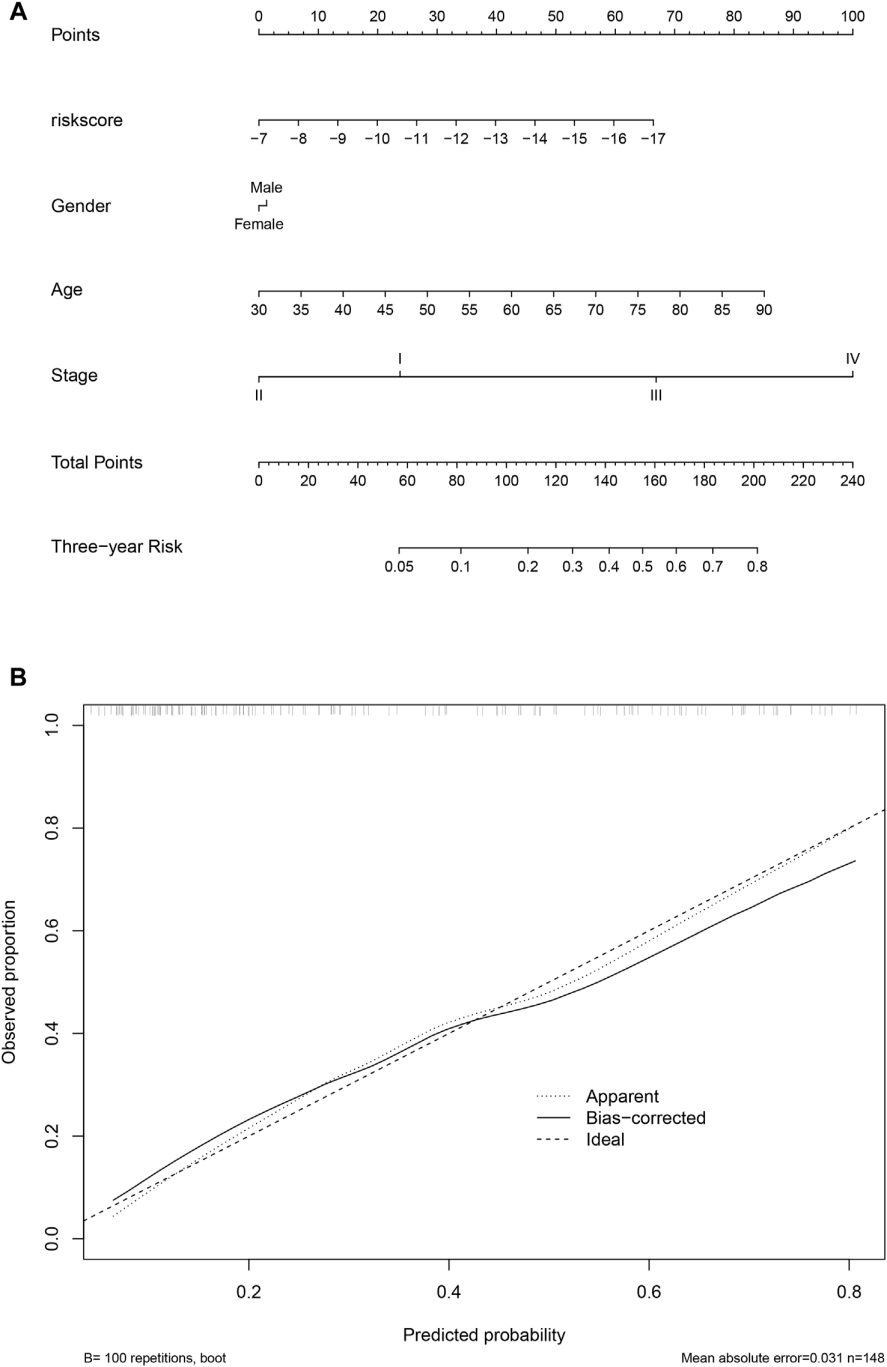
Nomogram and calibration. The nomogram considering clinical information and the score value was calculated and visualized **(A)**, and the calibration curve was also plotted **(B)**.

To facilitate the clinical utilization of the score, a nomogram simultaneously considering the score, gender, age, and stage was plotted. As shown in Figure 8A, clinical information contributed to the 3-year survival rate, and the score is among the most important indicators for the 3-year survival rate. A calibration curve is also plotted (Figure 8B).

### Candidate genes *UNG*, *SLC11A1*, and *LTB4R* promote proliferation

**FIGURE 9:**
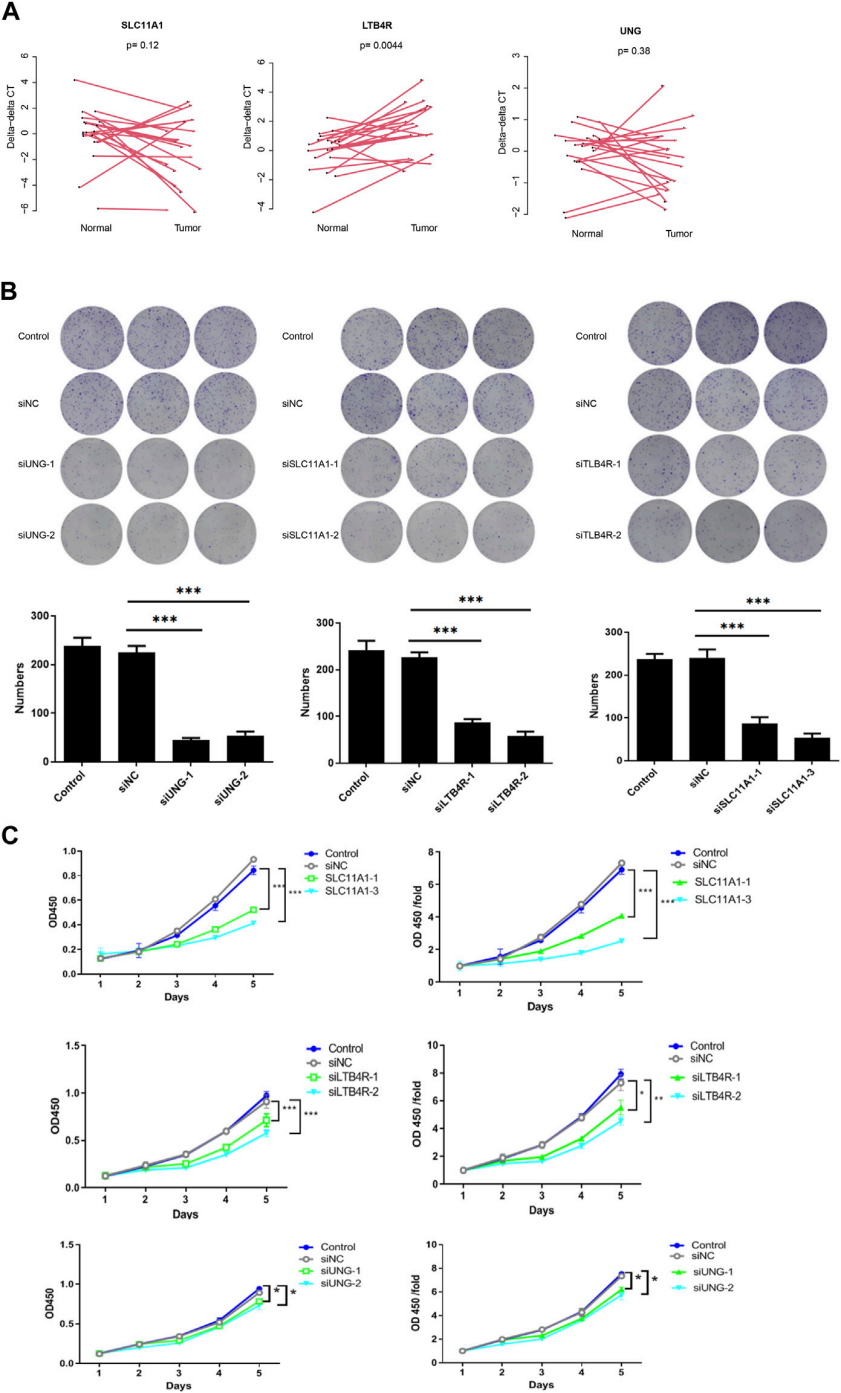
UNG, SLC11A1, and LTB4R promote proliferation in the RKO cell line. LTB4R was differentially expressed in 20 paired normal and cancerous tissues, while UNG and SLC11A1 were not **(A)**. Clone formation assay revealed that after the knockdown of UNG, SLC11A1, and LTB4R, clone formation significantly decreased **(B)**; the bottom panel is the bar plot showing the number of clones. The profanation of siUNG, siSLC11A1, and siLTB4R group was also significantly decreased **(C)**. *p <0.05, **p <0.01, and ***p <0.001.

To validate the function of the candidate genes, the expression of UNG, SLC11A1, and LTB4R in cancer and normal tissues was evaluated using qRT-PCR. Despite that, UNG and SLC11A1 were not differentially expressed, while LTB4R was (Figure 9A). After the knockdown of UNG, SLC11A1, and LTB4R in the RKO cell line, the proliferation of the comparison was seen using CCK8 and clone formation assay. As expected, the knockdown of UNG, SLC11A1, and LTB4R significantly decreased the clone formation rate (Figure 9B; *p* <0.05). Consistently, the knockdown of these genes also causes a decreased proliferation rate, according to CCK8 assay (Figure 9C; *p* <0.05). Collectively, these results suggest that the candidate genes impact the proliferation of the NSCLC cell line.

### Candidate genes *UNG*, *SLC11A1*, and *LTB4R* promote migration and invasion

**FIGURE 10:**
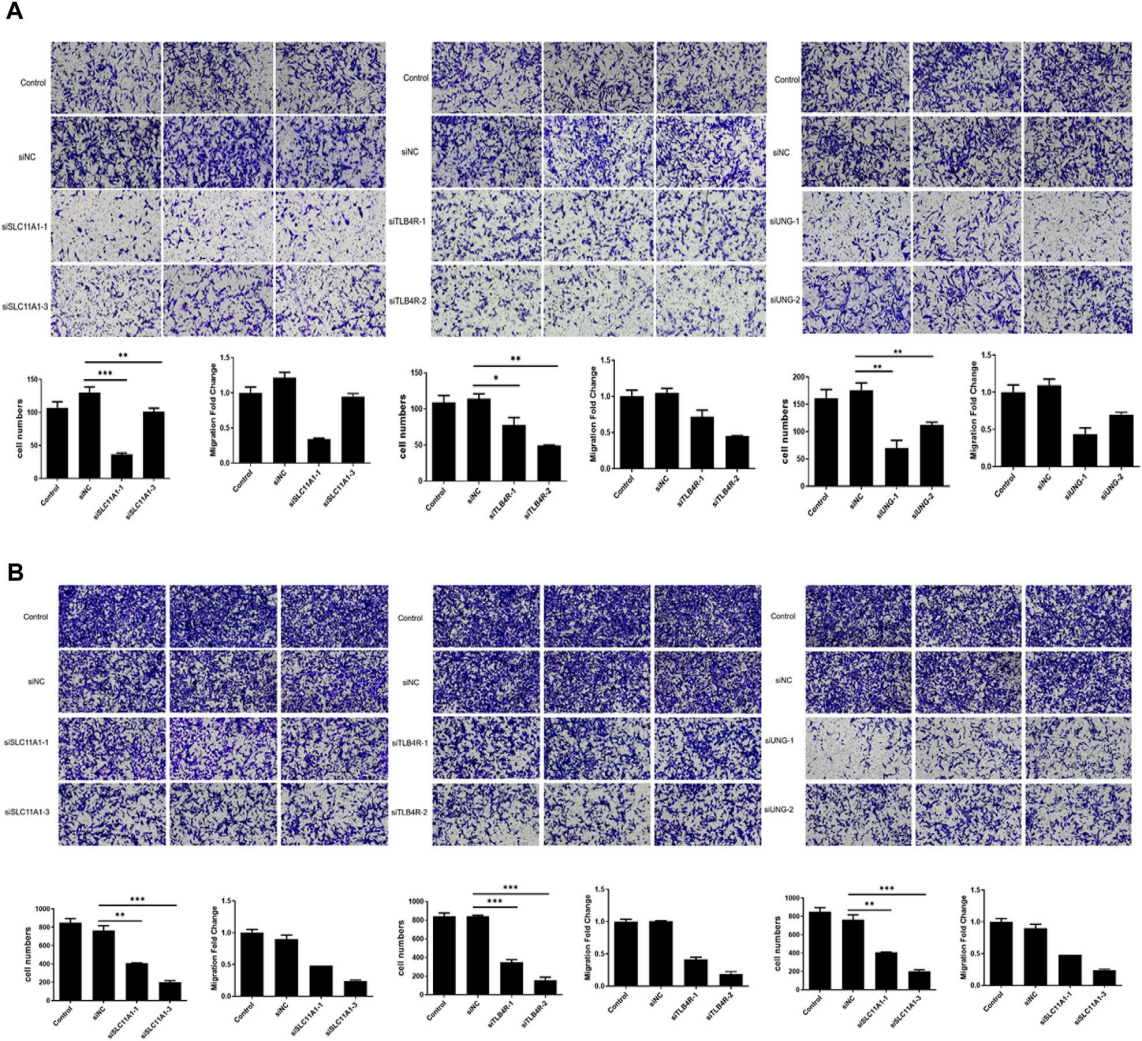
Migration and invasion significantly decreased after UNG, SLC11A1, and LTB4R knockdown. The migration **(A)** and invasion **(B)** rate was significantly decreased using the transwell assay.

Furthermore, migration and invasion assays were performed to evaluate the impact of UNG, SLC11A1, and LTB4R. The migration and invasion rate was quantified using the transwell assay. As shown in Figures 10A,B, the migration rate of RKO cell lines significantly decreased after the knockdown of these genes, compared to the control groups (*p* <0.05), indicating that these genes were involved in NSCLC development.

## Materials and methods

### Candidate gene identification and model development

The gene expression profile of TCGA, GSE39582 (Marisa et al., 2013), GSE17536, GSE17537 (Smith et al., 2010), GSE33113 (Kemper et al., 2012), GSE38832 (Tripathi et al., 2014), and GSE37892 (Laibe et al., 2012) was retrieved from cBioPortal (https://www.cbioportal.org/) and the Gene Expression Omnibus (GEO), respectively, according to the accession number. The TCGA mRNA expression value was converted into log_2_-transformed RSEM + 1 while the GEO datasets demonstrated the log_2_-transformed signal intensity. The corresponding clinical information, especially survival information, was downloaded along with the gene expression profile. After normalization, the correlation between the gene expression value and the overall survival was analyzed. After dividing it into high-expression and low-expression groups according to the median expression value of each gene, the survival difference between groups was evaluated, and the genes significantly associated with survival were retained as list1. The same analyses were performed on the GSE39582 dataset, and the genes were retained as list2. To ensure that the genes function with the immune response, the “GOBP_IMMUNE_RESPONSE” gene list from MSigDB (Liberzon et al., 2015) (https://www.gsea-msigdb.org/gsea/msigdb) was retrieved as list3. The intersected genes were used as candidate genes for model development. In other words, the candidate genes fulfill the following criteria: listed in the “GOBP_IMMUNE_RESPONSE” item and significantly associated survival in both TCGA and GSE39582 datasets.

After the aforementioned candidate gene identification, 14 genes were retained. To search the global optimum solution, all possible combinations of these 14 genes were listed. A Cox multivariate regression model was developed according to the selected genes in TCGA dataset. The model was a linear model described as follows:

$$\sum_{i=1}^{n}b_{i}x_{i},$$

where x_i_ refers to the gene expression value of gene *i*, while b_i_ indicates the coefficient calculated according to the gene expression value and survival information. The best combination in both TCGA and GSE39586 datasets was used as the global optimized panel.

### Model performance evaluation in the training and test datasets

In each dataset, the score was calculated according to the same coefficients (b_i_), and the samples in each dataset were divided into low-/high-score samples, according to the median value of the corresponding dataset. The survival difference (including overall, recurrence-free, disease-free, metastasis-free, and progression-free survival) was analyzed between high-/low-score samples, in the training and testing datasets. The detailed survival and gene expression values were plotted using the R package “pheatmap”. The detailed information on each dataset (gene expression values, risk score values, and survival information) is provided in Supplementary Table S1.

### Genetic association

Gene mutation, copy number variation, and DNA methylation status in TCGA dataset were retrieved from cBioPortal. Differentially mutated genes between groups were evaluated using Fisher’s exact test (only non-synonymous mutation used), and significantly different (*p* <0.05) mutations were identified. The mutation status of each sample was visualized using the R package “GenVisR” (Skidmore et al., 2016). The differential CNV and DNA methylation sites were identified using the Wilcoxon test and R package “ChAMP” (Tian et al., 2017), respectively.

### GO and GSEA analyses

Differentially expressed genes were identified according to the samples in low-/high-score sample groups, using the R package “limma” (Ritchie et al., 2015). The differentially expressed genes were identified as an adjusted *p*-value <0.01 and |log2 fold change| >0.5. Afterward, enriched pathways were identified using the R package clusterProfiler:enrichGO (Yu et al., 2012). Ontology categories were set as “BP,” “MF,” and “CC,” respectively. For GSEA, clusterProfiler::GSEA was used according to the fold change of the genes between high-/low-score samples. The enriched pathways or sets were visualized using the R package “enrichplot”.

### Immune cell infiltration, drug sensitivity prediction, and nomogram

TCGA dataset was used for cell infiltration analyses. After transforming the gene expression values into TPM values, immune cell infiltration was evaluated with TIMER2 (Li et al., 2020) (http://timer.cistrome.org/), simultaneously using algorithms such as CIBERSORT (Newman et al., 2015), xCELL (Aran et al., 2017), EPIC, and MCP-counter (Becht et al., 2016). The infiltration difference between low-/high-score samples of each immune cell type was analyzed and visualized using the R package “vioplot”.

For drug sensitivity prediction, the R package “oncopredict” (Maeser et al., 2021) was used and the required dataset was retrieved. The IC_50_ values of all samples were calculated, and the drugs with significantly different IC_50_ values between low-/high-score samples were identified and visualized. The nomogram was calculated and visualized using the R package “rms,” and the calibration curve was also plotted.

### qRT-PCR, siRNA, and clone formation

The RNA of each sample was extracted using the TRIzol (15596026CN) reagent, according to the manufacturer-provided protocol. The primer is listed in Supplementary Table S2. The siRNA sequences used in this study are listed in Supplementary Table S3. The cells in each group in the logarithmic growth phase were digested using trypsin, suspended in the complete medium, and then counted using a cell counter. Inoculates of 800–1,500 cells per well (depending on the cell growth) were introduced in each group of a 6-well culture plate, with three replicates per group. The culture was placed in a CO_2_ incubator for 14 days or until the cell number in most single clones exceeded 50. The medium was replaced every 3 days, photographed using a fluorescence microscope, and washed once with PBS. Each well was fixed with 1 mL of 4% paraformaldehyde for 30–60 min, washed once with PBS, and then stained with a clean, impurity-free crystal violet staining solution (500 μL per well) for 10–20 min; then, it was washed several times with ddH_2_O, allowed to air dry, photographed, and the clone number was counted.

### CCK8 and transwell assay

Digest logarithmic growth phase cells using trypsin, suspend in a complete medium, and count the cells. Evaluate the cell density for each well based on the growth rate, and inoculate 100 μl of cells per well, with 3–5 replicate wells per group. After preparing the plates, allow the cells to settle completely, and observe the cell density of each group under a microscope before incubation. After 48 h, add 10 μL of the CCK8 reagent to each well, and do not change the medium until the end of the culture period. Incubate the cells for 1–3 h, shake the 96-well plate for 2–5 min using a shaker, and measure the OD value at 450 nm using an ELISA reader.

Add 100 µL of the serum-free culture medium to each 24-well plate chambers and place it in a cell culture incubator for 1–2 h. Digest the logarithmic cells with trypsin, suspend them in a low-serum culture medium, and count; carefully remove the culture medium from the chambers and add 600 µL of the culture medium containing 30% FBS to the lower chamber. Dilute the cells with the serum-free culture medium at a certain ratio, add 100 µL of the cell suspension (containing 100,000–200,000 cells) to each chamber, transfer the chambers to the lower chamber containing the culture medium with 30% FBS using forceps, and incubate it in a tissue culture incubator for 4–24 h. Remove the culture medium from the upper chamber and fix the cells with 4% paraformaldehyde (FPA) fixative at room temperature for 10–30 min. Remove the 4% FPA fixative and wash the cells in the upper chamber with 1 × PBS 1–2 times. Immerse the chambers in a staining solution for 5–10 min, and transfer the cells to the underside of a membrane for staining. Invert the chambers onto an absorbent paper to remove the culture medium, and gently remove the non-transferred cells with a cotton swab, wash the upper chamber several times with ddH_2_O, air dry, and photograph under a microscope.

## Discussion

The clinical demand for a prognosis for colorectal carcinoma promotes studies regarding cancer biomarkers. In the last few decades, single biomarkers have been widely reported and some biomarkers have been used in clinical practice, including TMB and MSI (Messersmith, 2019; Luo et al., 2021; Ashktorab and Brim, 2022). However, due to genetic heterogeneity and complex microenviromental interactions, the performance of single biomarkers is disappointing. Recently, multiple biomarker-based models, especially those based on transcriptome, have been emphasized, due to its robustness (Chen et al., 2019; Ecker et al., 2021; Lin et al., 2021). However, these studies have some limitations, among which the most important is the lack of totally independent or insufficient validation sets. For example, Hang et al. (Zheng et al., 2022) developed a CRC model using stem cell-related genes and assayed the performance of the model, but no totally independent dataset was used for validation. It is similar in another study using immune cycle genes (Hou et al., 2022). Dagui et al. used a validation set, but only one was used (Lin et al., 2021). The highlight of multiple gene-based models is the robustness, but the aforementioned study ignored it, which may result in overfit. Our study used 1,325 samples across seven datasets from different countries and centers, generated using different platforms (including RNA-seq and microarray), indicating the robustness of the model.

Among the genes in the model, it was noticed that PAK1 was reported to induce liver metastasis using ERK (Li et al., 2010) and was required for cell proliferation (Qing et al., 2012). LTB4R was associated with proliferation by inducing apoptosis (Ihara et al., 2007). ICOS, SPFQ, and FSTL1 were also reported to be correlated with proliferation, cell adhesion, and prognosis (Zhang et al., 2016; Gu et al., 2018; Meng et al., 2022), while the studies were focused on gene polymorphism of LEP and CASP6 (Choi et al., 2012; Liu et al., 2014). Notably, SLC11A1 was reported as a potential biomarker for prognosis and immune therapy efficiency (Ma et al., 2022). These reports suggest that the model based on these genes may reflect various statuses of colorectal cancer. It is notable that the genes were immune-related genes, according to MSigDB, and SLC11A1 was reported to be associated with the microenvironment (Ma et al., 2022) in CRC and a similar result was found for CASP6 in gliomas (Guo et al., 2022). The other genes were also reported to reflect or influence the immune status of various types of cancers (Boggio et al., 2022). Our results indicate that a high proportion of immune cells were significantly associated with the model, which is consistent with the previous studies.

There are several limitations to this study. First, despite the fact that the samples were from different centers, they are still retrospective samples. Important clinical indicators are not available. Thus, a double-blind study is still necessary before clinical utilization. Second, pooled data (to generate the best cutoff value) are needed for each platform. The datasets used different platforms, and this study used a median value as the cutoff, but in clinical practice, the optimized cutoff value is necessary. In addition, drug sensitivity and immune infiltration are calculated using algorithms based on the transcriptome, which may have brought a bias. Lastly, detailed functions of genes of the model need further investigation.

## Data Availability

The datasets presented in this study can be found in online repositories. The names of the repository/repositories and accession number(s) can be found in the article/Supplementary Material.
