# Supplementary figures and images for: Immune-related gene-based model predicts the survival of colorectal carcinoma and reflected various biological statuses
Source: Front Mol Biosci. 2023 Oct 18;10:1277933. doi: 10.3389/fmolb.2023.1277933 (PMC10619740; doi:10.3389/fmolb.2023.1277933)

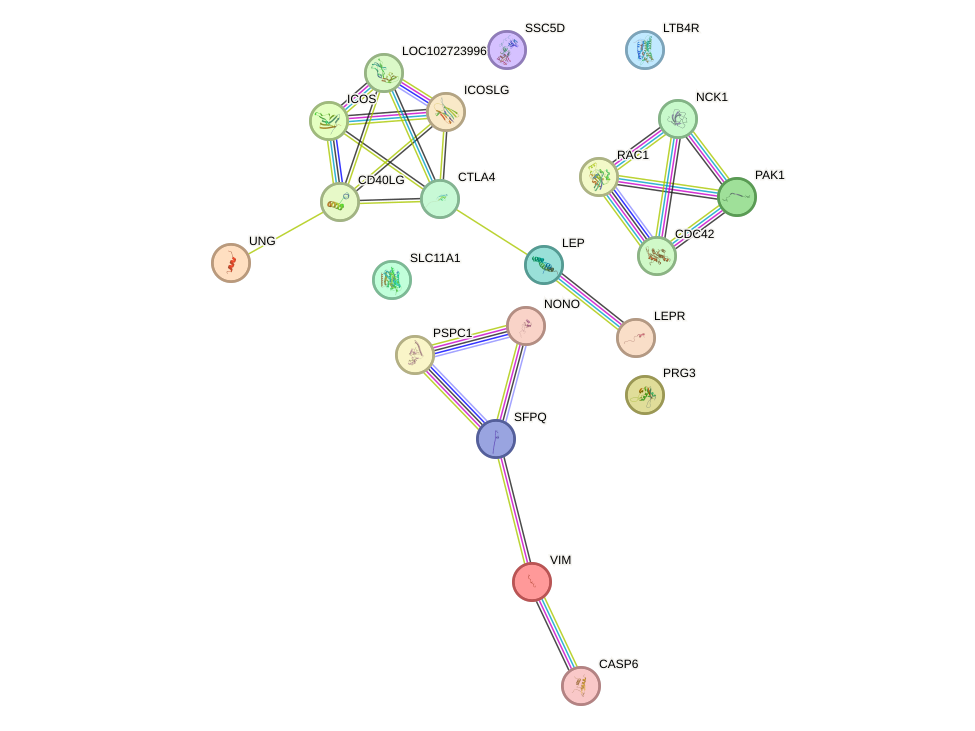

Supplement: Supplementary file 4 [file Image1.PNG]
